# Genetic and biological characterizations of a Newcastle disease virus from swine in china

**DOI:** 10.1186/1743-422X-9-129

**Published:** 2012-07-02

**Authors:** Xiaoyuan Yuan, Youling Wang, Jinxing Yang, Huaiying Xu, Yuxia Zhang, Zhuoming Qin, Hongbin Ai, Jinbao Wang

**Affiliations:** 1 College of Life Science, Shandong Normal University, Jinan, 250014, People's Republic of China; 2 Shandong Academy of Agricultural Sciences, Jinan, 250023, People's Republic of China

**Keywords:** NDV, Swine, Genetic and biological characterizations, F gene

## Abstract

**Background:**

Newcastle Disease Virus (NDV) has been considered to only infect avian species. However, one paramyxovirus named as Xiny10 was isolated from swine. The differences of Xiny10, another previous swine NDV (JL01) and vaccine strain La Sota were compared on the basis of sequences of the whole-lengthen Fusion (F) gene and biological characteristics.

**Findings:**

Through serologic tests and sequence alignment, Xiny10 was proved as NDV. It has great differences with JL01 in virulence, biological characteristics, genotype and amino acid homology of F gene. The sequence alignment showed Xiny10 and La Sota both belonged to genotype II. It shared 97.3% to 98.7% identities with genotype II NDVs, which was higher than these strains from the other genotypes.

**Conclusions:**

These above data suggested that the swine virus was NDV and it might be generated from La Sota.

## Introduction

NDV or avian paramyxovirus type I (APMV1) is one of the most serious infectious agents affecting poultry, and it can cause serious economic losses in poultry [1,2]. In the past few years, NDV appeared frequently in China [3]. NDV has been considered to only infect avian species. However, one paramyxovirus isolate (Xiny10) was isolated from one sick swine whose clinical signs were characterized by progressive weight loss, fever and diarrhea in post-weaned pigs of approximately 9 weeks of age.

In this paper, Xiny10 and 24 other NDV isolates from different hosts in 1970–2010 were characterized molecularly, which would help us understand the potential relationship between swine NDV and the other NDVs from different hosts.

## Methods

Xiny10 was isolated from swine in Henan province of China in 2010. The sequences of field isolates and vaccine strains were downloaded from Genbank of NCBI, detailed in Table 1. The name rule for field isolates was Host. Location. Name. Year(Genbank No.). The cross-hemagglutination inhibition test, intracerebral pathogenicity index (ICPI), mean death time (MDT) and intravenous pathogenicity index (IVPI) were conducted as described in the OIE manual [4]. Animal maintenance and experimental protocols were approved by the Animal Experiment Ethics Committee of Shandong Academy of Agricultural Sciences.

**Table 1:** Properties of NDV strains in the study

**Strain**	**Abbr.**	**Strain**	**Abbr.**
La Sota(AF077761) #	La Sota	Australia.AUS32(M24700)	AUS32
B1(AF309418)	B1	Goose.Jiangsu.JS05.03(DQ363532)*	JS05
Bingham(A03663)	Bingham	Chicken.Shandong.SBZ.02(DQ227245)*	SBZ
Herts.33(AY741404)	Herts33	Chicken.Argentina.TL.70(AY734534)	TL70
F48E9(AY508514)	F48E9	Ostrich.Guilin.TN.07(EF589138)*	TN07
TEX-48(M24698)	TEX-48	Layer.GuiZhou.FW.07(EF589135)*	FW07
Taiwan95(U62620)	TW95	Penguin.China.BJ.99(DQ227250)*	BJ99
Queenland.V4(AF217084) #	V4	Muscovyduck.China.FP1.02(FJ872531)*	FP1
NIreland.Ulster.67(AY562991)	Ulster67	Duck.Shandong.SY.03(DQ228923)*	SY03
Turkey.USA.VGGA.89(AY289002)	VGGA	Pigeon.China.PB.96(DQ858355) *	PB
Gamefowl.US.211472.02(AY562987)	211472	Swine.China.Xiny.10(JN032760)*	Xiny10
Anhinga.USA.44083.93(AY562986)	44083	Swine.China.JL01.07(EF464163)*	JL01
Parrot.India.CUL.97(AY359876)	CUL97		

“#”represents the most popular vaccine isolate in China now;

“*”represents these isolates are from different hosts in China.

Xiny10 strain was isolated from swine’s liver by using 10-day-old SPF chicken embryonated eggs. The viral RNA was extracted from the cultures of embryo by using the MiniBEST viral RNA Extraction kit (TaKaRa, Japan). The one-step RT-PCR was performed with NDV-specific primers designed by the F gene [GenBank: AF077761]. F-F: 5′-atg ggc tcc aaa cct tct ac-3′, and F-R: 5′-ttg tag tgg ctc tca tc-3′, targeting 1662 bp. The thermal profile of RT-PCR was 50°C for 40 min and 94°C for 2 min, followed by 35 cycles of 94°C for 90s, 52°C for 90s, 72°C for 90s, and a final extension cycle of 72°C for 10 min. The fragment was cloned and sequenced by Shanghai BGI company and subsequently submitted to NCBI [Genbank: JN032760]. Nucleotide sequences and phylogenetic tree were analyzed by the MegAlign program (DNAStar Inc. Madison) and MEGA4.0 (http://www.megasoftware.net).

## Findings

Through serologic tests and sequence alignment, Xiny10 was proved as NDV. F protein cleavage site of NDV is an important determinant of NDV pathogenicity and virulence [5,6]. The F protein cleavage site showed Xiny10 had a common motif ^112^ G-R-Q-G-R-L^117^ that was consistent with lentogenic vaccine virus La Sota. And the biological tests suggested that Xiny10 possessed separately the MDT of 102 h, the ICPI of 0.2, the IVPI of 0, which proved it was highly lentogenic. Interestingly, as reported experimentally [7], another swine NDV JL01 was an lentogenic strain with F protein cleavage site ^112^ G-K-Q-G-R-L^117^, but the biological virulence was velogenic.

**Figure 1:**
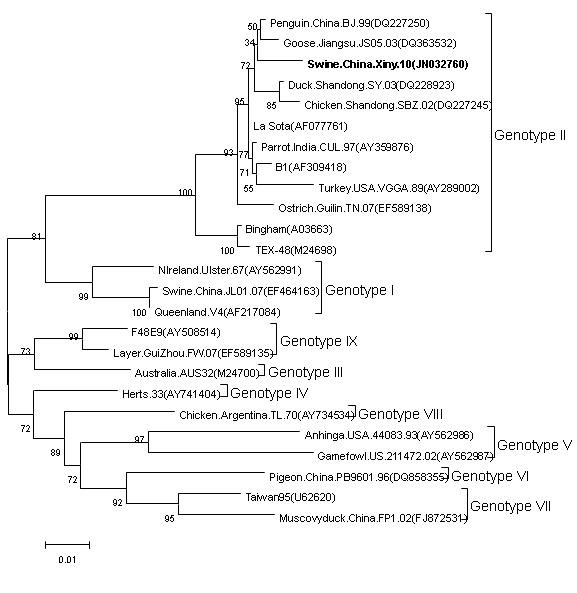
**Phylogenetic tree of the amino acid sequences of 25 NDV strains based on F gene.** The phylogenetic tree was generated by the neighbor-joining method with MEGA4.0.

From the phylogenetic tree of the amino acid sequences [8,9] (Figure 1), Xiny10 and vaccine strain La Sota belonged to genotype II, but JL01 and V4 belonged to genotype I. And La Sota and V4 are the most popular vaccines in China now. The comparison of amino acid sequences showed Xiny10 and JL01 shared 98.7%, 92.8% identities with La Sota; and the homology between the two isolates was only 91.7%. In summary, these data confirmed that Xiny10 strain had great differences with previously reported swine strain in virulence, genotype, and amino acid homology of F gene.

Among all genotype II NDVs, some isolates were derived from chicken, duck, goose and other poultry varieties, but there were also isolates from wild birds, such as: turkey (VGGA89), penguin (BJ99), parrot (CUL97). As one of them, Xiny10 shared 97.3%–98.7% identities with genotype II NDVs, which was higher than 91.9% for genotype I(Ulster67,V4), 90.6% for genotype III(AUS32), 91.9% for genotype IV(Herts33), 87.3% for genotype V(44083,211472), 87.3% for genotype VI(PB), 88.1%–89.2% for genotype VII(TW95,FP1), 90.1% for genotype VIII(TL70), 91.1%–91.3% for genotype IX(F48E9,FW07).

## Discussions

We confirmed the paramyxovirus isolate Xiny10 from swine was NDV. Furthermore, it was obtained from one area where swine and poultry mixed for breeding. Meanwhile, it had such a high amino acid homology with vaccine La Sota. In addition, Xiny10 and La Sota owned the same 12 conserved Cysteine residues and 6 potential glycosylation sites which maybe affect viral structure and antigenicity[10]. Additionally, Xiny10 revealed the higher amino acid homology with the field strains from different hosts of genotype II. These above data suggested that the swine virus might be generated from La Sota because it has been widely used as live virus vaccine until now in China.

## Competing interests

The authors declare that they have no competing interests.

## Authors’ contributions

XY, YW, JY, HX, YZ, ZQ carried out these experiments and wrote the manuscript. HA, JW participated in experimental design and coordination. All authors read and approved the final manuscript.

## References

[B1] Alexander D Newcastle disease and other avian paramyxoviruses Revue scientifique et technique (International Office of Epizootics) 2000 19 443 462 10935273 10.20506/rst.19.2.1231

[B2] Hanson RP Brandly C Newcastle disease Ann N Y Acad Sci 1958 70 585 597 10.1111/j.1749-6632.1958.tb35414.x 13559920

[B3] Yu L Wang Z Jiang Y Chang L Kwang J Characterization of newly emerging Newcastle disease virus isolates from the People’s Republic of China and Taiwan J Clin Microbiol 2001 39 3512 3519 10.1128/JCM.39.10.3512-3519.2001 11574565 PMC88381

[B4] Alexander DJ Manual of diagnostic tests and vaccines for terrestrial animals 2004 Volume 5 Paris 270 282

[B5] Deng R Wang Z Mirza AM Iorio RM Localization of a domain on the paramyxovirus attachment protein required for the promotion of cellular fusion by its homologous fusion protein spike Virology 1995 209 457 469 10.1006/viro.1995.1278 7778280

[B6] Toyoda T Sakaguchi T Imai K Inocencio NM Gotoh B Hamaguchi M Nagai Y Structural comparison of the cleavage-activation site of the fusion glycoprotein between virulent and avirulent strains of Newcastle disease virus Virology 1987 158 242 247 10.1016/0042-6822(87)90261-3 3576973

[B7] Ding Z Cong Yan-long Chang S Wang Guang-mei Wang Z Zhang Quan-peng Hao Wu Sun Yu-zhang Genetic analysis of avian paramyxovirus-1 (Newcastle disease virus) isolates obtained from swine populations in China related to commonly utilized commercial vaccine strains Virus Genes 2010 3 369 376 20661635 10.1007/s11262-010-0516-1

[B8] Lomniczi B Wehmann E Herczeg J Ballagi-Pordany A Kaleta E Werner O Meulemans G Jorgensen P Mante A Gielkens A Newcastle disease outbreaks in recent years in western Europe were caused by an old (VI) and a novel genotype (VII) Arch Virol 1998 143 49 64 10.1007/s007050050267 9505965

[B9] Czegledi A Herczeg J Hadjiev G Doumanova L Wehmann E Lomniczi B The occurrence of five major Newcastle disease virus genotypes (II, IV, V, VI and VIIb) in Bulgaria between 1959 and 1996 Epidemiol Infect 2002 129 679 688 10.1017/S0950268802007732 12558353 PMC2869932

[B10] Chambers P Millar NS Emmerson PT Nucleotide sequence of the gene encoding the fusion glycoprotein of Newcastle disease virus J Gen Virol 1986 67 2685 2694 10.1099/0022-1317-67-12-2685 3025345

